# Biometry and Intraocular Lens Power Calculation by Combined Scheimpflug-Placido Disc versus Optical Interferometry Devices

**DOI:** 10.18502/jovr.v17i4.12349

**Published:** 2022-11-29

**Authors:** Mehlan Juliane, Lehman Anne-Isabel, Cichocki Myriam, Druchkiv Vasyl, Katz Toam, Stephan J Linke

**Affiliations:** ^1^Department of Ophthalmology, University Medical Center Hamburg-Eppendorf (UKE), Hamburg, Germany; ^2^Zentrumsehstärke, Hamburg, Germany; ^3^CareVision Hamburg, Germany; ^5^https://orcid.org/0000-0003-0677-6937

**Keywords:** Cataract Surgery, IOL Calculation, Ray Tracing

## Abstract

**Purpose:**

To compare the results of the current gold standard, laser interferometry, and keratometry by the IOL-Master, with a newly developed Galilei G6 using raytracing software Okulix for intraocular lens (IOL) power calculations.

**Methods:**

For comparison of the IOL-power calculation of both devices, we analyzed the difference between the actual one-month postoperative subjective refraction and the theoretically calculated target refraction before cataract surgery. The IOL was selected according to the IOL Master recommendation aiming for emmetropia after surgery.We analyzed the differences of the measurements of the basic biometric data in 205 healthy eyes by each device.

**Results:**

Our study included 205 healthy, unoperated eyes from 117 patients (61 women, 56 men) aged 20 to 75 years. Twenty-two eyes of cataract patients were also included in this retrospective study design. The mean difference between the prediction of the postoperative refraction and the refraction actually achieved was 0.03 D for the IOL Master and –0.23 D for the Galilei G6. The difference was not statistically significant (*P* = 0.059). The difference between the IOL power calculation of the IOL Master and the calculation of the G6 was not statistically significant (*P* = 0.064). The difference between the predicted refraction of the G6 and the refraction achieved after one month was also not statistically significant (*P* = 0.12) and neither was the difference between the predicted refraction of the IOL Master and the achieved refraction (*P* = 0.39). The mean axial length was calculated as 24.21 ± 0.80 mm using the IOL Master and 24.27 ± 0.82 mm using the Galilei G6 device. The mean value regarding anterior chamber depth (ACD) of the IOL master was 3.46 ± 0.23 mm and for the Galilei was G6 3.51 ± 0.25 mm. When comparing the white to white (WTW) values of the IOL master, it showed mean values of 12.32 ± 0.31 and Galilei showed mean values of G6 12.21 ± 0.28. All of these differences (between Galileo and IOL Master measurements) were statistically significant (*P* < 0.001).

**Conclusion:**

Both the laser interferometry/keratometry performed by the IOL Master and the interferometry/raytracing biometry strategy performed by the Galilei G6 demonstrated equal results when executing the IOL power calculation before cataract surgery in eyes with no prior ocular surgery.

##  INTRODUCTION

Cataract surgery is the most often performed operation worldwide with an estimated number of 32 million procedures in 2020 according to the World Health Organization. Precise measurement of the dimensions of the eye and the consecutive calculations are especially essential to guarantee a reliable determination of the intraocular lens (IOL) power. To further increase the precision of the measurement, new devices incorporating tomography (anterior and posterior corneal surfaces) have been developed.

A widespread device for IOL power calculation is IOL Master 500 (Zeiss, Germany) which uses partial coherence interferometry for the axial length and lens thickness and placido and Scheimpflug technology for the k values and calculates the IOL-power using the established formulas such as Haigis, SRK/T, etc.

Ziemer Galilei G6 (Ziemer Ophthalmic Systems AG, Port, Switzerland) is an ocular biometry device combining Scheimpflug and Placido analyses for detailed corneal analyses and A-scan optical interferometry (880 nm wavelength) for optical measurement of all parts of the eye from the anterior corneal surface to the retina which corresponds with the axial length.

In addition to the standard IOL-calculation formulas, Galilei G6 has the option to use an additional (external) IOL-power calculation software called OkulixⓇ which uses ray-tracing measurements and calculations for a potentially more precise IOL power calculation. Three different ray-traced IOL power calculation approaches are used: The first calculation named “parax” focuses on the calculation of paraxial light rays. This calculation model is focused on the almost unrefracted light rays entering the eye near the optical axis.

The second calculation which is called “best focus” takes into account light rays over a pupil diameter of 2.5 mm to determine the IOL power. The third calculation approach is referred to as the total refraction calculation which provides the sphero-cylindrical IOL power which takes into account the corneal anterior and posterior surfaces.

This study was designed to compare the combined Scheimpflug/Placido and optical A-scan interferometer biometer (Galileo G6, [Ziemer Ophthalmic Systems AG, Port, Switzerland]) with the previous gold standard, a partial coherence interferometer biometer (IOL Master 500, Zeiss, Germany).

##  METHODS

Our study included 205 healthy, unoperated eyes from 117 participants (61 women, 56 men) aged 20 to 75 years. Only subjects without ocular pathology or prior ocular surgery were included in this study.

### Part I

For our first analysis, the power of the implanted IOL for the 22 cataract patients (triLisa 1st Q or Alcon SA60AT) was determined according to the IOL Master recommendation using the Haigis formula while aiming for post-surgical emmetropia.

The prediction for the postoperative spherical equivalent (SE) of the IOL Master 500 (partial coherence interferometry + distance-independent telecentric keratometry) using the Haigis formula was compared to the prediction of the Galilei G6 Systems using the Okulix ray tracing formula for the same IOL. We decided to evaluate the data after one month because of uncomplicated cataract surgery and the refraction could be expected to stabilize after one month.^[9]^

To compare the accuracy of the IOL-power calculation of both devices, first, we analyzed the difference between the actual one-month postoperative subjective refraction and the preoperative theoretically calculated target refraction. Pre- and postoperative measurement of the uncorrected and corrected distance visual acuity (UDVA & CDVA) was also performed. Postoperative examinations were performed one month after the operation.

### Part II

In addition to the IOL power and target refraction measurements, we compared the following parameters that were also calculated by each device: axial length (AL), white-to-white (WTW), and anterior chamber depth (ACD).

Measurements of the keratometry were not compared directly with each other because the devices use different procedures and measuring zones. The IOL Master uses a distance-independent telecentric keratometer for the keratometry and the keratometry is measured at 32 points in two concentric rings (diameter 1.65 and 2.3 mm) while the Galilei G6 uses simulated keratometry data that is measured using placido-based corneal topography.

IOL Master 500 was at the moment of this retrospective study the gold standard for calculating the appropriate power of the intraocular lenses for successful cataract surgery. Its axial length measurement is based on the application of partial coherence interferometry. Diagnostic limitations of this device include mature cataract, central scarring of the cornea and epiretinal membranes.

Anterior chamber depth is measured from the anterior corneal surface to the anterior lens surface using the Scheimpflug principle. This is only possible with phakic eyes, otherwise the rear border is missing. A measurement of the anterior depth chamber is only possible if the corneal radii have been calculated before executing the Scheimpflug method.

WTW measures the horizontal diameter of the iris and the deviation of the visual axis from the center of the iris. The range of the measurement is usually from 8 to 16 mm.

For measuring the axial length, a range between 14 and 39 mm is specified. The measuring range of the keratometer is 5 to 10 mm. Measurement of the anterior chamber depth is possible in the range of 1.5 to 6.5 mm. Scaling for all three measured variables takes place in 0.01 mm increments.

Galilei G6 combines the technologies of Placido topography, Dual-Scheimpflug tomography, and optical biometry. For measuring the axial length, a range between 14 and 40 mm is specified. The measuring range of the keratometer is 4.5 to 13.5 mm. Measurement of the anterior chamber depth is possible in the range of 1.5 to 6.5 mm.

Galilei G6 enables the measurement of the anterior and posterior surfaces of the cornea, as well as the thickness of the cornea and the thickness of the lens.

The ocular ray tracing allows for a calculation of the refraction based on Snell's law. Snell's law describes the change in the direction of propagation of a plane wave when it transitions into another medium. The reason for the change in direction is the change in the material-dependent phase velocity, which is defined and represented as the refractive index. Rays can be calculated for any distance from the optical axis and for other parameter variations. There are three different evaluation modes for the Galilei G6 – parax, best-focus, and total refraction calculation. These modes will only be briefly explained here in order not to go beyond the scope of this manuscript.

The rating mode "parax" means the paraxial IOL power. This is the calculation that best focuses the light rays on the retina. The best focus calculation also considers light rays above a pupil diameter of 2.5 mm to calculate the IOL. The total refraction calculation means the spherocylindrical IOL power, which takes into account the anterior and posterior corneal surfaces. Only the best focus analysis was used for this evaluation.

### Statistical Analysis 

Distribution of differences between the refraction achieved and predicted by Galilei and the IOL master was illustrated using the Bland–Altman plots. Differences were determined by the “Limits of Agreement" and their confidence intervals summarized. The 95% limits of agreement show where 95% of the differences can be expected. One can contrast the theoretical values against the confidence intervals of these limit values and thus assess the level of repeatability. The mean and its confidence interval are also on the graph to show the possible orientation of the differences between the achieved and the predicted measurements. Other visualization methods were also used. On the one hand, we illustrated the achieved and predicted refraction in the predefined intervals using a bar chart and showed the results ungrouped using the box plots. The differences in distribution between the achieved and predicted refraction were checked using the non-parametric Wilcoxon sign test. Bland–Altman graphics were used again for the repeatability of the measurements for AL, WTW, and ACD calculated. The influence of the escaping cases was prevented by utilizing the Yuen test for trimmed differences used. The two tests dealt with in the conclusion are the mentioned non-parametric Wilcoxon sign test and the Yuen test.

**Figure 1:**
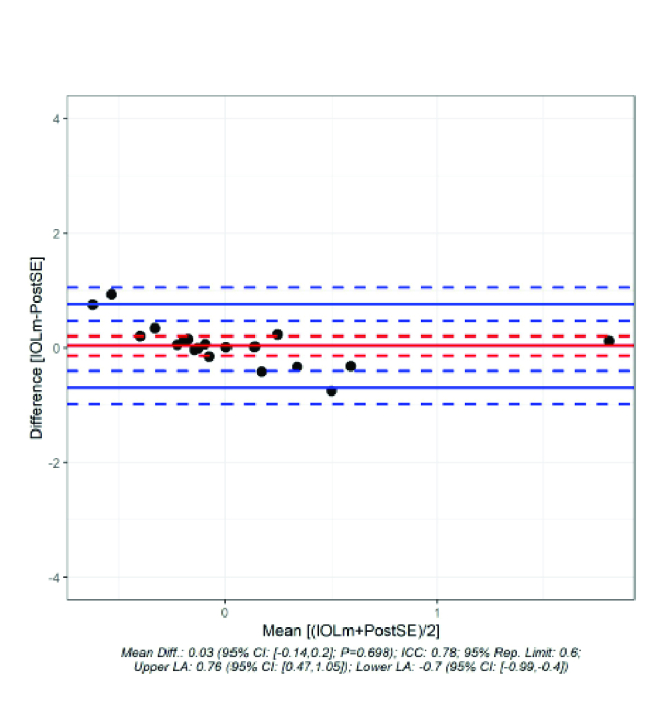
Bland–Altman Plot to visualize the difference of the IOLM to post OP SE.

**Figure 2:**
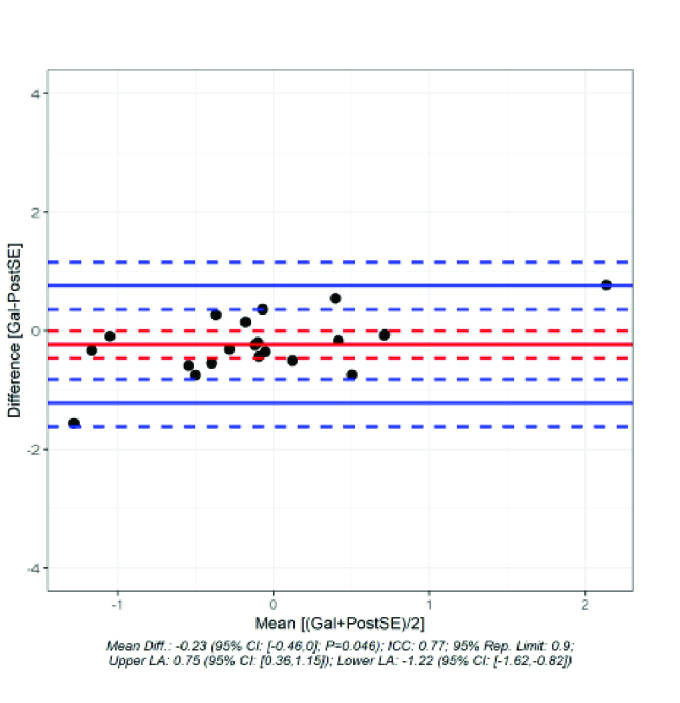
Bland–Altman Plot to visualize the difference of the G6 theoretical calculation post OP SE if lens calculated with G6.

**Figure 3:**
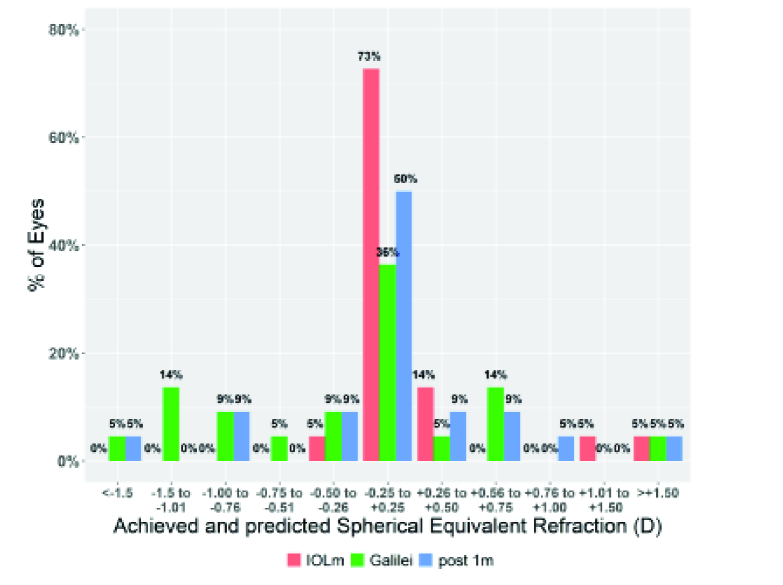
Comparison of achieved and predicted spherical equivalent.

**Figure 4:**
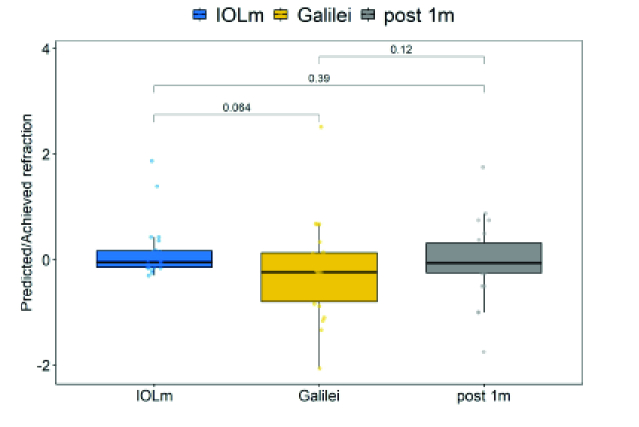
The quotient of the predicted and achieved refraction is shown.

**Table 1:** Biometric standard data.


**Factors**	**Range (Min / Max)**	Mean ± SD*
AL	
Galilei	29.95 / 31.69	24.27 ± 0.82
IOL – Master	20.94 / 31.64	24.21 ± 0.80
Difference	-1.56 / 0.45	-0.05 ± 0.03
P- Wert	< 0.001	
ACD	
Galilei	2.31 / 5.49	3.51 ± 0.25
IOL – Master	2.31 / 4.68	3.46 ± 0.23
Difference	-0.81 / 1.26	-0.05 ± 0.05
P- Wert	< 0.001	
WtW	
Galilei	11.23 / 13.02	12.21 ± 0.28
IOL – Master	11.50 / 13.40	12.32 ± 0.31
Difference	-0.77 / 1.01	0.12 ± 0.06
P- Wert	< 0.001	
	
	
Al, axial length; IOL, intracocular lens; ACD, anterior chamber depth; WtW, white-to- white; SD, standard deviation *Trimmed means with Winzorized Standard Deviations		

##  RESULTS

### Part I

Twenty-two eyes (eight male and seven female patients) were operated by the same experienced surgeon using phacoemulsification.

The mean difference between the prediction of the postoperative refraction and the refraction actually achieved was 0.03 D for the IOL Master [Figure 1]. In contrast, the difference was –0.23 D for the Galilei G6 [Figure 2]. The difference between the prediction of refraction and actual achieved refraction for IOL Master and Galilei was not statistically significant (*P* = 0.059). Even if one looks at the standard deviation (IOL Master: 0.371 D; G6: 0.504 D), the difference was not statistically significant. The Pitman–Morgan test was not significant for both variances (*P* = 0.189).

Figure 3 shows the predicted postoperative refraction values for IOL Master and Galilei G6, as well as the actual postoperative values.

Calculation with the IOL Master predicted that 92% of the cases would be in the emmetropic range. Calculation with the Galilei G6 system using ray tracing, after matching the IOL strength and the IOL model, predicted 50% in the emmetropic range. The range from –1.0 D to +1 D was predicted with a 92% probability by the IOL master and 91% by the Galilei G6 system.

Half of the postoperative results, using the IOL power calculation based on the IOL Master biometry, ranged from –0.25 D to 0.25 D, 68% of the postoperative results were in the range of –0.50 D to +0.50 D (emmetropia) and 91% of the results ranged from –1 D to +1 D.

Figure 4 shows the results of the comparison of the predicted refraction of the IOL Master and the Galilei G6 and the results of the refraction actually achieved one month after the operation.

Difference between the calculations of the IOL Master and the calculations of the G6 were not statistically significant (*P* = 0.064).

Difference between the predicted refraction of the G6 and the refraction achieved after one month was also not statistically significant (*P* = 0.12) and neither was the difference between the predicted refraction of the IOL Master and the achieved refraction (*P* = 0.39).

The box plot shows a tendency toward a wider spread of the values measured with the Galilei G6. However, there is a tendency toward a myopic refractive shift when calculating with the Okulix ray tracing software.

### Part II – Comparison of the Biometric Data

We compared the calculations for the axial length, anterior chamber depth and white-to-white measured with the Galilei G6 and the IOL master of all 205 eyes. Due to different procedures and measuring zones in each device we did not compare the measurements of the keratometry directly with each other (see above).

Evaluation of the biometric standard data in Table 1 shows a mean axial length of 24.21 ± 0.80 with the IOL Master and 24.27 ± 0.82 with the Galilei G6. The difference was statistically significant (*P* < 0.001). There was also a significant difference in the ACD (*P* < 0.001). The mean value for the IOL Master was 3.46 ± 0.23 and for the Galilei G6 was 3.51 ± 0.25.

When comparing the WTW values of the IOL master which showed mean values of 12.32 ± 0.31 with that of the Galilei G6 which revealed mean values of 12.21 ± 0.28, these differences were again statistically significant (*P* < 0.001).

In general, there was a statistically significant longer axial length, deeper anterior chamber, and a steeper cornea in the measurements of the Galilei G6 as compared to the IOL Master.

##  DISCUSSION

In the first part of this study, the prediction for the postoperative SE of the IOL Master 500 using the Haigis formula was compared to the prediction of the Galilei G6 Systems using the Okulix ray tracing formula for the same IOL. The mean difference between the IOL Masters' predicted values in comparison to the actually achieved postoperative SE is 0.032 D. The Galilei G6 using ray tracing demonstrated a mean difference pre-surgical calculation to post-surgical subjective refraction SE of –0.234 D.

The calculations using the Okulix ray tracing software seem to have a slight tendency toward a myopic shift. Before treatment, the IOL-Master calculation (Haigis formula) predicted that 92% of the cases would be in the emmetropic range. However, only 68% of the operated eyes reached the emmetropic range postoperatively (–0.5 dpt to +0.5 dpt). Calculation with the Galilei G6 system using ray tracing predicted 50% in the emmetropic region after matching the IOL power and the IOL model. If you look at the range from –1.0 D to +1 D, then 92% of the calculations with the IOL Master and 91% of the calculations with the Galileo reach the range.

An exact IOL calculation is essential for a reliable and precise outcome when performing cataract surgery and phacoemulsification. The main hurdle in terms of reaching target refraction at the moment is the difference between the estimated and effective lens/IOL position (Li et al; 2019). In an effort to have a more accurate calculation for the IOL to be selected, many parameters of the optical system should be included in the calculation formula of the IOL.

Currently the lens calculation using the IOL Master is the gold standard for preoperative lens calculations. However, new devices that are capable of retrieving additional parameters are emerging, which include Galilei G6, and other systems such as IOL Master or Cassini, which include Galilei G6 and other systems such as Cassini. In our study, the IOL power was calculated using the IOL Master 500.

Regarding Figure 4, the box plot shows a tendency toward a wider spread of the values measured with the Galilei G6. However, there is a tendency toward myopic shift when calculating with the Okulix ray tracing software. You should keep this in mind when choosing lenses and pay attention accordingly.

Despite the noticeable differences between the predictive values of the two measurement devices versus the actually achieved postoperative refraction calculated for the same IOL, there was no statistically significant difference between the two devices (*P* = 0.059).

Preoperative correction planning had a positive impact on the discrepancies between the actually achieved postoperative SE and the predicted target refractions from both the IOL Master and Galilei G6. It can cause the actual refraction values to be closer to the predicted values. With regard to astigmatism, it is important to mention that a postoperative astigmatism value reduced to –1.0 dpt was consciously tolerated. Should it be more, the implantation of a toric IOL was discussed with the patient.

A postoperative reading for astigmatism reduced down to –1.0 D was deliberately tolerated. If it occurred beyond that, the implantation of a toric IOL was discussed with the patient.

If the patient prefers not to wear varifocal glasses after the operation, there is also the option of performing an additional LASIK procedure postoperatively or performing limbal relaxing incisions during the initial operation to reduce the postoperative astigmatism.

Preoperative correction planning is now part of everyday clinical practice and has been modified so that a postoperative astigmatism of > –0.5 D would be avoided.

Ghoreishi et al reported similar results in 2018.^[2]^ They compared the IOL Okulix ray tracing software with the SRKT and Hoffer Q formula on 104 patients with cataract. In this instance, the IOL was also selected using the IOL Master 500. They could not find a statistically significant difference when comparing the Okulix ray tracing software with the other two formulas (*P* = 0.25).

Ventura et al also showed that there were no significant differences in their calculations between the IOL Masters 500 and the Galilei G6 system.^[8]^ The recommended lens power was calculated for each patient using the Haigis formula for the Acrysof SN60WF IOL. In the IOL calculation for postoperative emmetropia there was no statistically significant difference (*P* = 0.49) between the predictive calculation and the actual postoperative calculation.

If we look at the differences in the calculation between the IOL Master and the Galilei G6 in terms of the SE achieved after one month, there are varying results. The difference between calculation of the IOL Master and calculation of the G6 is not statistically significant (*P* = 0.064).

The difference between the predicted refraction of the G6 and the refraction achieved after one month is also not statistically significant (*P* = 0.12) and neither is the difference between the predicted refraction of the IOL Master and the achieved refraction (*P* = 0.39).

In the second part of the current study, we compared the measurements of AL, ACD, and WTW from the IOL Master 500 and the Galilei G6 system. As there exists multiple measurement methods including different areas/zones regarding the K values, we have not compared them directly to each other. There are statistically significant differences between each of the measurements of AL, ACD, and WTW (*P* each < 0.001). These results partially contradict other studies.

Ventura et al^[8]^ measured 88 eyes with the IOL Master 500 and the Galilei G6 and could not find statistically significant differences regarding AL (*P* = 0.456), ACD (*P* = 0.468) or the *K*-values (average *P* = 0.432).

Lee et al^[4]^ did not find a significant difference between IOL Master 500 and Galilei G6 system when measuring the AL (*P* = 0.321). However, the measurements of ACD and *K*-values were statistically significantly different between the two devices (*P* < 0.001 and *P* = 0.028, respectively). The statistically significant difference regarding the *K*-values could again be due to different measurement methods. In summary they stated that the absolute prediction error for the IOL Master 500 and Galilei G6 regarding postoperative refraction is not significantly different (*P* = 423):^[4]^

In the study of Jung et al,^[3]^ the IOL Master 700 and Galilei G6 were compared. Both devices showed reliable repeatability, but the IOL Master 700 was slightly superior to the Galilei G6. There were no significant differences in axial length, anterior chamber depth, steepest K, white-to-white corneal diameter or lens thickness. However, the flat *K*-value and the central corneal thickness differed (*P* < 0.05).

The IOL Master 700, like the IOL Master 500, utilizes a distance-independent telecentric keratometer for keratometry measurements and the keratometry values are measured at 32 points that are arranged in two concentric rings with a diameter of 1.65 and 2.30 mm. The Galilei G6 system uses simulated keratometry data measured using placido-based corneal topography.^[3]^

The IOL Master 700 utilizes swept source technology in contrast to the IOL Master 500. Shajari M et al performed a study on 79 patients to analyze possible differences in the parameters used for lens calculation (axial length, corneal curvature, and anterior chamber depth). The Pentacam AXL (Scheimpflug technology with partial coherence interferometry), the IOL Master 700 (swept-source optical coherence tomography), and the IOL Master 500 (optical biometer) were all compared. In conclusion, they could state that there were no statistically significant differences regarding those parameters.^[9]^

Dalto et al described the differences between the SRKT and Haigis formulas in terms of preoperative factors and refractive outcomes.^[1]^ All eyes were implanted with the Alcon-SN60WF IOL and preoperative measurements were made with LENSTAR from Haag-Streit (optical biometry). Their research revealed that there were differences in lens recommendations for the two formulas. Eyes that had myopia and recorded lower *K*-values preoperatively had the possibility of a slight myopic shift with the Haigis formula. On the other hand, these eyes were predisposed to have a hyperopic shift with the SRKT formula.^[1]^

Furthermore, Zhu et al performed a comparison between the Haigis, SRKT, and the Holladay formulas with patients diagnosed with high myopic eyes. Their conclusion was that for these patients, the Haigis or SRKT formulas can reduce the errors in the IOL calculation where the Haigis formula is always preferable when *K* ≤ 43 D and the axis length is over 30 mm.^[8]^

It should be noted, however, that while it is mathematically possible for the target refraction to be ± 0.1 D, the art lenses are so far available only in 0.5 D increments and even with the same IOL from the same manufacturer, there can be subtle differences in refractive strength. Thus, the mathematical accuracy can still be difficult to implement in practice.

It should also be considered that patients' compliance has an impact on the results. Comorbidities, such as spinal or neck problems, ptosis and dermatochalasis or sicca also play a major role in determining the choice of IOL calculation.

In summary, there is no significant difference between the results of the ray tracing method of the Galilei G6 and the measurements derived from the IOL Master. Regarding the prediction of the postoperative SE for the respective IOL, we could not determine any inferiority of the Galilei G6 system as compared to the current gold standard of the IOL Master 500. The relevance for clinical use and also the possible benefits in eyes with previous operations or after refractive surgery should be evaluated in further studies.

##  Financial Support and Sponsorship

None.

##  Conflicts of Interest

None.
